# Liver injury: the therapeutic dilemma of homeopathy – a case report from Bangladesh

**DOI:** 10.1097/MS9.0000000000000918

**Published:** 2023-05-22

**Authors:** Abhigan B. Shrestha, Bikash Karki, Prakash Acharya, Shashwat Kafley, Nabaraj Shrestha, Shailendra Karki, Surya K. Acharya

**Affiliations:** a M Abdur Rahim Medical College, Dinajpur; b Enamn Medical College, Dhaka, Bangladesh; c Patan Academy of Health Sciences, Lalitpur; d Kathmandu Medical College, Kathmandu, Nepal; e Department of Internal Medicine, New York City Health and Hospitals, Woodhull Medical Centre, Brooklyn, New York, USA

**Keywords:** case report, homeopathy, liver injury

## Abstract

Despite the lack of scientific evidence supporting its effectiveness, homeopathic treatment is increasingly being used as a form of alternative medicine, with many people taking homeopathic remedies instead of drug therapies. It is based on the principle of ‘like cures like’, meaning that a remedy similar to the illness can be used to treat it. However, there have been several reports suggesting the risks of homeopathic remedies, among which homeopathy-induced liver injury is widely discussed. Here, we report a case of a 35-year-old well-oriented male patient with a typical clinical presentation of liver injury as presented by yellowish discoloration of sclera and skin along with generalized body itching following the use of homeopathic medicine for musculoskeletal pain. Laboratory reports of increased liver markers along with bilirubin were also suggestive. Excluding other differentials like viral hepatitis, alcoholic hepatitis, hemochromatosis, Wilson disease, and standard drug and toxin-induced hepatitis, the recent use of homeopathic remedies was a contributing factor in leading to the diagnosis of homeopathy-induced liver injury. He was then treated with the discontinuation of homeopathic medicine and supportive care. This case highlights the need for public awareness of the possible complications such as headache, tiredness, skin eruption, dizziness, bowel dysfunction, allergic reactions to acute pancreatitis, renal failure, neurological dysfunction, possible liver injury, and even mortality in those patients who pursue homeopathic treatments and health care professionals should take this into account when making a differential diagnosis in patients with liver injury.

## Introduction

Highlights

- Many people use homeopathy, despite its lack of scientific evidence of effectiveness.
- In this case, we highlight the role of homeopathy leading to drug-induced liver injury.
- Diagnosis can be done by taking a proper history and excluding other potential differentials.

Homeopathy is a form of alternative medicine that has been around for centuries, but its efficacy and safety have been widely debated. In general, the findings of systematic reviews of homeopathic trials have produced mixed results, with some meta-analyses reporting that homeopathic treatments for a variety of conditions are linked to positive results compared to placebo, while others show no difference compared to placebo^1^. Many people still choose to use homeopathic remedies to treat their ailments. There is evidence to support the use of homeopathy for certain conditions, such as anxiety and anxiety disorder, depression, insomnia^2–4,^ complementary therapy for fibromyalgia sufferers^5,^ and the use of these remedies as a treatment can result in consequences like allergic reactions, acute pancreatitis, neurological manifestations^6,^ severe liver injury in those with underlying liver disease and even mortality and morbidity^7^. This article has been defined according to the CAse REports (CARE) guideline^8^.

## Case presentation

A 35-year-old male with no significant past medical or family history presented with yellowish discoloration of the skin and sclera for 1 month. He also reports generalized body itching for the same duration. The patient is a married man and is strictly monogamous. He is non-alcoholic and has no history of any specific drug intake. Moreover, for the past 6 months, he has complained of musculoskeletal pain due to his work; he reports the use of homeopathic remedies based on alternate days since but could not provide the name of the specific medication.

**Figure 1:**
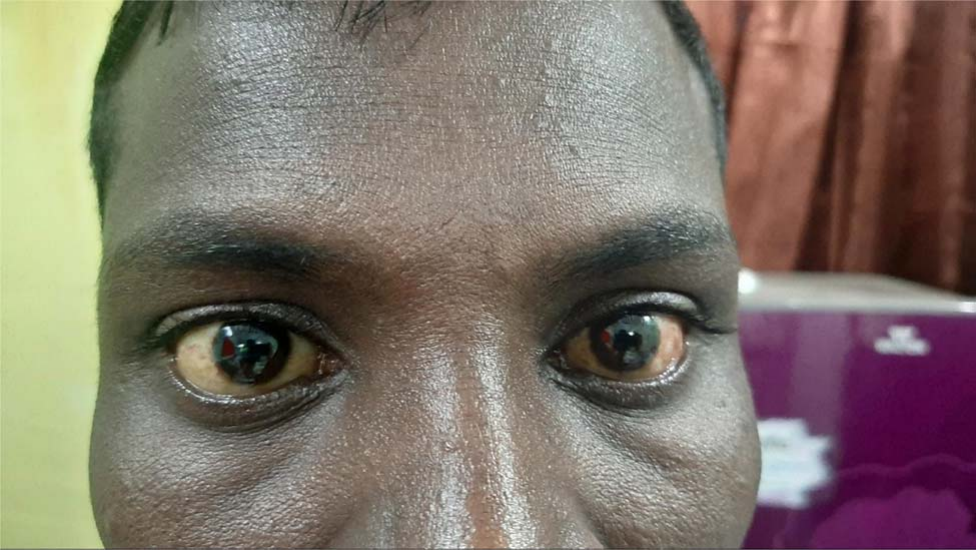
Bulbar sclera showing icteric.

On physical examination, he was of thin build, pale looking, tachycardiac (heart rate of 110 beats per minute) with low blood pressure (100/60 mmHg) and febrile with his recorded body temperature of 100°F. Skin examination showed excoriation over the body and bilateral scleral jaundice was observed. Presence of mild hepatomegaly on abdominal examination. His musculoskeletal examination showed no joint tenderness or swelling. The remainder of the cardiopulmonary and neurological examination shows a normal impression (Fig. 1 and Table 1).

**Table 1:** laboratory investigations of the patient.

Test	Result (normal values)
Hemoglobin (Hb)	9.5 g/dl (13.5–17.5 g/dl)
Total leukocyte count (TLC)	7000/mm^3^ (4000–11 000/mm^3^)
Neutrophils	78%
Lymphocytes	20%
Eosinophil	1%
Platelets	282 000
Peripheral blood smear	Normocytic normochromic anemia
LFT	
Total bilirubin	26.5 mg/dl
AST	140 U/l
ALT	639 U/l
ALP	350
Total protein	5.5
Gamma-glutamyl transferase	40 U/l
PT	13 s (11–13.5s)
Urea/creatinine	40/0.4
Urine analysis	Normal report
Urine culture	No growth
Blood culture	Negative
Iron profile	
Serum ferritin	>1500 ng/ml (adult male: 18.0–270.0 ng/ml)
Serum iron	60 µg/dl (50–150 µg/dl)
Serum transferrin saturation	40%
Serum TIBC	300 µg/dl (310–340 µg/dl)
ESR	50 mm/h (1–20 mm/h)
CRP	4 mg/dl (<5 mg/dl)
Uric acid	3.2 mg/dl (2.5–6 mg/dl)
Viral serology	
HIV	All negative
HBsAg	
Hepatitis C	
Hepatitis A IgM	
Hepatitis E IgM	
Rapid malaria test and dengue IgG/IgM	Negative

ALP, alkaline phosphatase; ALT, alanine transaminase; AST, aspartate transaminase; CRP, C-reactive protein; ESR, erythrocyte sedimentation rate; HBsAg, hepatitis B surface antigen; HIV, human immunodeficiency virus; Ig, immunoglobulin; LFT, liver function test; PT, prothrombin time; TIBC, total iron-binding capacity.

His lab results show hemoglobin of 9.5 g/dl and a total leukocyte count of 7000/mm^3^, with 78% neutrophils. A peripheral blood smear shows normocytic normochromic anemia. Liver enzymes were total bilirubin 26.5 mg/dl, AST (aspartate transaminase) 140 U/l, ALT (alanine transaminase) 639 U/l, ALP (alkaline phosphatase) 350 U/l, gamma-glutamyl transferase 40 U/l with total protein of 5.5 g/dl. The iron profile shows serum ferritin greater than 1500 ng/ml, serum iron 60 µg/dl, serum transferrin saturation 40%, and TIBC (total iron-binding capacity) 300 µg/dl. Levels of blood ESR (erythrocyte sedimentation rate), CRP (C-reactive protein), and uric acid were 50 mm/h, 4 mg/dl, and 3.2 mg/dl, respectively. His routine urine reports normal findings and the culture of urine and blood shows no growth of organisms. Viral serology (HIV, hepatitis A, B, C, and E), rapid malaria, and dengue immunoglobulin (Ig)G/IgM were negative.

Initial investigations revealed mild anemia (Hb 9.5 g/dl) with normal rest of the routine tests. Elevated liver markers (ALT 639 IU/l, ALP 350 IU/l, along with the significant rise in the level of bilirubin of 26.5 mg/dl) indicate a cholestatic pattern of liver injury. An increase in the level of inflammatory markers ESR 50 mm/h and an increased level of serum ferritin (>1500 ng/ml) with normal transferrin saturation also paved the way for acute inflammatory changes in the liver. The viral serological lines of hepatitis (A, B, C, and E), HIV, and common blood-borne illnesses like malaria and dengue were negative. Radiological findings revealed that the chest X-ray shows the normal report, and abdominal ultrasonography shows the presence of mild hepatomegaly along with normal ductal anatomy. The upper gastrointestinal endoscopy showed mild ulceration with no evidence of varices. 24-hour urinary copper and slit lamp examinations revealed normal findings.

After excluding differentials like viral hepatitis (negative viral serological marker), alcoholic hepatitis (no history of alcohol and acute radiological changes in the liver), Wilson disease (normal 24-hour urinary copper, slit lamp examination, along with no history of neuropsychiatric manifestation), hemochromatosis (high ferritin with normal transferrin saturation and negative family history), and the onset of clinical manifestation after starting homeopathy medication, a diagnosis of homeopathic induced liver injury was made.

There is no definitive therapy for this condition, so supportive therapy was commenced with oral ursodeoxycholic acid (UDCA) (150 mg once daily for 2 weeks), PPI, and sirup sucralfate with advice on prompt withdrawal of homeopathic medication. As many studies have highlighted, UDCA plays an essential role in cellular defense against apoptosis and oxidative stress and promotes cell survival by activating antioxidant cascades. Even high doses of UDCA are nontoxic to the liver. On 2 weeks of follow-up, his jaundice (total bilirubin: 18 mg/dl) and itching was significantly improved with a gradual decrease in his liver enzyme levels (ALT 320, AST 80 U/l). A plan to continue on the same oral medication for 8 weeks and follow-up was made with complete resolution of itching and his scleral sign of jaundice with near-to-normal bilirubin (total bilirubin: 3 mg/dl) and liver enzymes (AST: 60 U/l, ALT: 42 U/l levels). He has been regularly followed up to date.

## Discussion

Homeopathic medicines are made through the ‘potentization’ process, which involves serially diluting and ‘succussing’ the beginning material, which often exceeds a limit where little to no ingredients remain. A systematic review reported that up to 9.2% of adults in most Western countries have relied on homeopathic remedies during the past 12 months^9^. Moreover, its prevalence has been shooting in Asian countries too; a multicenter study in Bangladesh highlights the prevalence of homeopathic medicine as the most prevalent type of treatment among conventional medicine users (52.2%)^10^. The majority of the people who used these medications had self-treated with over-the-counter items. This highlights the importance of awareness that needs to be significantly addressed. A systematic review of the published case reports and case series on the adverse impact of homeopathy revealed various events, such as allergic and immune-mediated reactions and the toxic effect of the potential content of formulations. On top of that, some of the homeopathic medicine also are adulterated with steroids^11^. While steroids are effective in treating certain conditions, they also have significant potential side effects and must be used with caution.

We are not blaming homeopathy directly; however, with respect to all the medical fields equally, it is also important to acknowledge the potential benefits and limitations. The most frequent conditions treated with homeopathic remedies were respiratory and otolaryngological (18.5%), musculoskeletal (12.3%), fatigue, sleep problems, stress or chronic pain (7.7%), gastrointestinal (5.0%), neurologic (3.4%), and mental health (2.1%). However, there are reports of potential health adverse effects like allergic reactions, severe acute pancreatitis, neurological manifestations^6,^ severe liver injury in those with underlying liver disease, and even mortality and morbidity^7^.

The diagnosis of any drug-induced liver injury (DILI) is based on excluding the differentials, whereas some experts define liver damage as evident by an isolated increase in levels of AST ×5 times above the upper limit of normality (ULN), AST 3 times over the ULN associated with the elevation of total bilirubin ×3 times above the ULN, or the increase of ALP above 2 times the ULN^12^. However, the beginnings of liver damage may arise more than 6 months after exposure to some drugs, drug-induced liver injury often manifests within 6 months of starting a new drug^7^.

Patients may remain asymptomatic in most cases; the pertinent features in patients with homeopathy-induced liver injury included jaundice at index presentation in all and ascites notable in one-third^6^. Similar to our case, the patient presented with clinical manifestation of jaundice and had been on homeopathy medicine for 6 months to overcome his pain manifesting abnormal liver parameters (ALP 639, AST 140, ALP 350) within the line of acute inflammation as evidenced by the rise in inflammatory marker like ESR (50 mm/h) and ferritin (>1500 ng/ml) levels. To assess the severity and monitor for acute liver failure, early detection of toxicity is crucial.

Evaluation of a patient with abnormal liver tests must start with a detailed medical history, any possible exposure to hepatotoxins (such as alcohol and medications), whether the patient is at risk for viral hepatitis, whether they have other conditions that are linked to the liver disease (Wilson or hemochromatosis), or a potential predisposing condition. On the floor of evaluating our top differentials, our patient is non-alcoholic with normal neurological manifestation on examination, and lab parameters reveal negative viral serological marker of hepatitis (A, B, C, E) and HIV along with normal 24-h urinary copper excretion and slit lamp examination of the eye shows normal findings that help in excluding the differentials.

There is no definitive treatment; withdrawal of the harmful toxin is the cornerstone of HILI (homeopathy-induced liver injury) therapy, and symptomatic management aids in recovery. UDCA plays an essential role in cellular defense against apoptosis and oxidative stress or promotes cell survival by activating antioxidant cascades. Also, to expedite the resolution of cholestatic DILI, the use of UDCA acid has successfully been applied^13^.

However, the patient did not bring the homeopathy drug (chemical composition), which was the major limitation of our case report.

## Conclusion

In the context of homeopathy, some people swear by its effectiveness, while others remain skeptical. It is important to acknowledge that homeopathy has its benefits; however, due to lacking scientific evidence, homeopathy also has its limitations. This case report emphasizes uplifting awareness among homeopathic users due to its adverse health effects, including liver injury that can result in fatal and chronic transformation in no time. Health workers should exclude all other potential differentials before making this diagnosis, as the actual disease may masquerade, and timely intervention and management with strict follow-up should be aimed accordingly.

## Ethical approval

Not applicable.

## Patient consent

Written informed consent was obtained from the patient for the publication of this case report and accompanying images. A copy of the written consent is available for review by the Editor-in-Chief of this journal on request.

## Sources of funding

Not applicable.

## Author contribution

All authors contributed equally.

## Conflicts of interest disclosure

There are no conflicts of interest.

## Research registration unique identifying number (UIN)

Not applicable.

## Guarantor

Abhigan Babu Shrestha.

## Data availability statement

Not applicable.

## Provenance and peer review

Not commissioned, externally peer-reviewed.

## References

[1] Shang A Huwiler-Müntener K Nartey L . Are the clinical effects of homoeopathy placebo effects? Comparative study of placebo-controlled trials of homoeopathy and allopathy. Lancet 2005;366:726–732. 16125589 10.1016/S0140-6736(05)67177-2

[2] Pilkington K Kirkwood G Rampes H . Homeopathy for anxiety and anxiety disorders: a systematic review of the research. Homeopathy 2006;95:151–162. 16815519 10.1016/j.homp.2006.05.005

[3] Pilkington K Kirkwood G Rampes H . Homeopathy for depression: a systematic review of the research evidence. Homeopathy 2005;94:153–163. 16060201 10.1016/j.homp.2005.04.003

[4] Cooper KL Relton C . Homeopathy for insomnia: a systematic review of research evidence. Sleep Med Rev 2010;14:329–337. 20223686 10.1016/j.smrv.2009.11.005

[5] Boehm K Raak C Cramer H . Homeopathy in the treatment of fibromyalgia – a comprehensive literature-review and meta-analysis. Complement Ther Med 2014;22:731–742. 25146079 10.1016/j.ctim.2014.06.005

[6] Stub T Kristoffersen AE Overvåg G . Adverse effects in homeopathy. A systematic review and meta-analysis of observational studies. Explore (NY) 2022;18:114–128. 33303386 10.1016/j.explore.2020.11.008

[7] Chalasani N Bonkovsky HL Fontana R . United States Drug Induced Liver Injury Network, Features and outcomes of 899 patients with drug-induced liver injury: the DILIN prospective study. Gastroenterology 2015;148:1340–1352.e7. 25754159 10.1053/j.gastro.2015.03.006 PMC4446235

[8] The CARE Guidelines: Consensus-based Clinical Case Reporting Guideline Development – PMC. Accessed 27 March 2023. https://www.ncbi.nlm.nih.gov/pmc/articles/PMC3833570/

[9] Relton C Cooper K Viksveen P . Prevalence of homeopathy use by the general population worldwide: a systematic review. Homeopathy 2017;106:69–78. 28552176 10.1016/j.homp.2017.03.002

[10] Shahjalal M Chakma SK Ahmed T . Prevalence and determinants of using complementary and alternative medicine for the treatment of chronic illnesses: a multicenter study in Bangladesh. PLoS One 2022;17:e0262221. 34986159 10.1371/journal.pone.0262221 PMC8730415

[11] Gupta SK Kaleekal T Joshi S . Misuse of corticosteroids in some of the drugs dispensed as preparations from alternative systems of medicine in India. Pharmacoepidemiol Drug Saf 2000;9:599–602. 11338919 10.1002/pds.553

[12] Aithal GP Watkins PB Andrade RJ . Case definition and phenotype standardization in drug-induced liver injury. Clin Pharmacol Ther 2011;89:806–815. 21544079 10.1038/clpt.2011.58

[13] Robles-Díaz M Nezic L Vujic-Aleksic V . Role of ursodeoxycholic acid in treating and preventing idiosyncratic drug-induced liver injury. A systematic review. Front Pharmacol 2021;12:744488. 34776963 10.3389/fphar.2021.744488 PMC8578816

